# The Role of Self-Objectification and Physical Exercise in Social Appearance Anxiety and Restrained Eating Among Female College Students

**DOI:** 10.3390/bs15101300

**Published:** 2025-09-23

**Authors:** Chang Hu, Wen Zhang, Wenying Huang

**Affiliations:** 1 School of Physical Education, Jiangxi Normal University, Nanchang 330027, China; huchang@itc.ynu.edu.cn (C.H.); cxks050829@163.com (W.Z.); 2 School of Physical Education, Yunnan University, Kunming 650091, China

**Keywords:** social appearance anxiety, self-objectification, restrained eating, physical exercise, female college students, moderated mediation model, eating behavior

## Abstract

This study aims to explore the relationship and underlying mechanisms between social appearance anxiety (SAA) and restrained eating (RE) among female college students. Although previous studies have shown a correlation between SAA and RE, the internal pathways remain unclear. Based on social comparison theory and objectification theory, this study constructs a model that includes self-objectification (SO) as a mediating variable and physical exercise (PE) as a moderating variable to analyze the impact mechanisms of SAA on RE. Adopting a cross-sectional online survey design, this study collected data from 2161 female college students in China. The results showed that SAA was significantly positively related to SO (*β* = 0.37, *p* < 0.001) and RE (*β* = 0.34, *p* < 0.001). SO partially mediated the relationship between SAA and RE (*β* = 0.125, 95% CI [0.104, 0.149]). Moreover, PE moderates the relationship between SAA and RE (*β* = −0.15, *p* < 0.001). However, PE’s moderating effect is insignificant in the relationship between SO and RE. These findings offer valuable insights and suggest that interventions targeting SAA and SO, as well as promoting PE, may help improve RE among female college students.

## 1. Introduction

Restrained eating (RE) consciously restricts calorie intake to lose or maintain weight (Polivy et al., 2020). Eating disorders are considerably more prevalent in females, with female college students representing a particularly vulnerable population (Niu et al., 2020; Song et al., 2019; Yao et al., 2021). Globally, approximately 9% of the U.S. population experiences an eating disorder during their lifetime, with higher prevalence among women than men. Studies in Australia show that 10% of female college students report engaging in restrained eating (Silén & Keski-Rahkonen, 2022; Streatfeild et al., 2021). In Asia, research indicates that the prevalence of restrained eating among Chinese female college students is as high as 62.0%, a rate significantly higher than in other groups (Chen & Jackson, 2008). This prevalence is closely related to the idealized body aesthetics promoted through social media and the appearance comparison pressures within campus environments (Ren et al., 2024; Yong et al., 2021; Zhang et al., 2018). In China, the prevalence of SAA among college students is particularly pronounced due to the widespread use of social media and the pervasive influence of popular culture (Xian et al., 2024). Chinese social media platforms are predominantly characterized by an aesthetic standard emphasizing thinness, fairness, and youthfulness (Y. Liu & Li, 2024), which has significantly heightened college students’ focus on and anxiety related to their appearance (Y. Wu et al., 2024). Online activities such as beauty rankings and weight-loss challenges exacerbate these concerns through constant comparison (M. Huang & Zhao, 2023). This constant comparison leads to SAA, resulting in RE behavior as students strive to achieve the idealized appearance (W. Huang et al., 2025a). Moreover, the extensive promotion of weight-loss and beauty products by Chinese fashion media and advertisements further strengthens the impact of SAA on college students’ eating behavior (Liao et al., 2023; L. L. Liu et al., 2018).

Restrained eating not only leads to physical issues such as malnutrition and weakened immunity (Habib et al., 2023; Morales et al., 2023) but also triggers psychological disorders like low self-esteem, anxiety, and depression, and may even increase the risk of suicide (Norwood et al., 2019; Stocks et al., 2019; Willmott et al., 2024). Therefore, exploring the mechanisms underlying restrained eating in female college students is critically important for developing targeted intervention strategies. Although previous research has identified a correlation between social appearance anxiety (SAA) and restrained eating, the underlying pathways remain unclear (Cañas et al., 2021). Social Comparison Theory and Objectification Theory provide a theoretical foundation for understanding this phenomenon. However, existing studies have not fully explored the mediating role of self-objectification (SO) and the moderating role of physical exercise (PE). SO and PE significantly impact an individual’s mental health (Martín-Rodríguez et al., 2024; Tiggemann & Slater, 2015). SO tends to contribute to negative body image and low self-esteem (Saunders et al., 2024), while PE has been shown to enhance body esteem and self-worth, potentially mitigating the adverse effects of SO (Ouyang et al., 2020). Therefore, this study aims to fill this gap by examining how SO and PE influence the relationship between SAA and restrained eating among female college students.

## 2. Literature Review and Hypotheses Development

### 2.1. Relationship Between Social Appearance Anxiety and Restrained Eating

The Social Comparison Theory posits that individuals frequently evaluate themselves against others in social contexts (Dijkstra et al., 2008; Gerber et al., 2018). Such comparisons can trigger SAA, a key psychological factor driving people to engage in RE (Brosof & Levinson, 2017; Caner et al., 2022). In the modern context, characterized by pervasive media and social media platforms, individuals are constantly exposed to idealized representations of physical appearance, including ultra-thin body types and flawless facial features. When individuals juxtapose their own appearance with these idealized images, they are prone to experiencing heightened anxiety regarding their physicality (Azem et al., 2023; Deng & Jiang, 2023). Empirical research has demonstrated that engaging in appearance-related comparisons on social media platforms can substantially intensify appearance-related anxiety (Czubaj et al., 2025; de Valle et al., 2021), which in turn may prompt individuals to resort to restrained eating to approximate the idealised appearance (Tian et al., 2024). For instance, studies have revealed that after women are exposed to fitness model images on social media, they experience increased appearance-related anxiety and are more inclined to embark on diets or other forms of restrained eating to alter their body shape (Cataldo et al., 2022; Rodgers et al., 2020; Vandenbosch et al., 2022). Moreover, cross-cultural investigations have also established a robust correlation between SAA and RE across diverse cultures (Alleva et al., 2015; Mitchison & Mond, 2015). This relationship may be especially salient in certain Eastern cultures, where considerable emphasis is placed on physical appearance (Eiring et al., 2021; Izydorczyk et al., 2021; Jenkinson et al., 2023).

### 2.2. The Mediating Role of Self-Objectification

SO refers to the tendency for individuals to perceive their bodies mainly from the perspective of an external observer, accompanied by ongoing monitoring and evaluation of their appearance (Daniels et al., 2020; Salomon & Brown, 2021; Schaefer & Thompson, 2018). Drawing on objectification theory (Fredrickson et al., 1998; Szymanski & Henning, 2007), when people are treated or perceived as sexual objects, they are more likely to experience heightened SAA (Veldhuis et al., 2020). This intensified SAA is not merely a temporary emotional reaction but rather establishes conditions for the development of SO (Baceviciene et al., 2022). In other words, recurrent experiences of SAA can gradually foster self-objectification, as individuals increasingly focus on how their bodies are judged by others (Sarda et al., 2025).

This process typically emerges during adolescence, when young people become especially invested in managing their body image, and it can give rise to psychological difficulties and maladaptive behaviors such as disordered eating. The media plays a crucial role in sustaining these dynamics by disseminating appearance-centered ideals (Fox et al., 2021; Maheux et al., 2021). Exposure to such media imagery fosters SAA, which subsequently promotes higher levels of SO. Experimental and correlational studies consistently show a strong association between SAA and SO across different ages, genders, races, and sexual orientations (Harsey & Zurbriggen, 2021; Schaefer et al., 2018; Skowronski et al., 2021; Zheng et al., 2018). Recent studies have further revealed that SO is influenced by SAA and serves as a mediator through which SAA contributes to individuals’ involvement in unhealthy behaviors (Hanna et al., 2017).

### 2.3. The Moderating Role of Physical Exercise

As the field of sport psychology has advanced, research shows that PE enhances mood, facilitates cognitive development, and reduces the risk of mental health issues (S. Biddle, 2016; S. J. H. Biddle et al., 2019; Lubans et al., 2016; Mahindru et al., 2023). Exercise typically energizes individuals, fostering greater vitality and positivity, which helps alleviate negative emotions (Lee et al., 2025; Ruiz-Ranz & Asín-Izquierdo, 2024; White et al., 2023). SAA is one such negative emotion, and it is plausible that exercise can help to alleviate this type of anxiety (W. Huang et al., 2025a). Moreover, PE may also play a role in mitigating the impact of SO on RE. Self-objectification emphasizes constant appearance monitoring, which often fosters restrained eating as a strategy to fit societal appearance ideals. However, engaging in exercise can redirect individuals’ focus from appearance concerns to bodily functionality, thereby reducing the reliance on RE as an appearance-regulation strategy. Empirical studies have found that individuals who engage in regular exercise tend to be more open-minded and cognitively focused (Dores et al., 2021; Hurst et al., 2017; L. Liu et al., 2024), while also experiencing greater positive emotions that buffer against maladaptive responses to stress (Ramos-Sanchez et al., 2021).

The integrative model of athletic performance underscores that positive mental experiences during PE, such as thoughts, emotions, and physical sensations, safeguard an individual’s mental and physical wellbeing (Anderson & Shivakumar, 2013; Herbert, 2022; Mandolesi et al., 2018). The protective-protective model (Zimmerman et al., 2013) posits that when influencing an outcome, protective factors can interact via two mechanisms: the enhancement hypothesis and the exclusion hypothesis (Fergus & Zimmerman, 2005). The enhancement hypothesis posits that two protective factors can strengthen their combined effect synergistically. Conversely, the exclusion hypothesis suggests that one protective factor can diminish the influence of another. Individuals who engage in frequent exercise are less susceptible to SAA’s effects on RE than those who exercise less. Consistent with this framework, individuals who engage in frequent exercise are also less susceptible to the negative effects of SO on RE. For example, adolescents who exercise regularly are better equipped to cope not only with the stress associated with SAA but also with appearance-related pressures linked to SO (Reigal et al., 2020). In contrast, those with low levels of exercise lack these protective benefits and are more vulnerable to adopting RE as a maladaptive coping strategy (Fietz et al., 2015; Li et al., 2024). Moreover, regular exercise enhances adaptive cognitive reappraisal strategies (Perchtold-Stefan et al., 2020), which facilitate the regulation of appearance-related stress and anxiety, thereby reducing reliance on RE.

### 2.4. The Present Study

In summary, this study proposes the following hypotheses for female college students:

**Hypothesis 1.**  *There is a significant association between SAA and RE.*

**Hypothesis 2.**  *SO is a mediator between SAA and RE.*

**Hypothesis 3.**  *PE can moderate both the direct link between anxiety and eating behaviors, as well as the second half of the mediating pathway involving SO, forming a moderated mediation model.*

**Figure 1:**
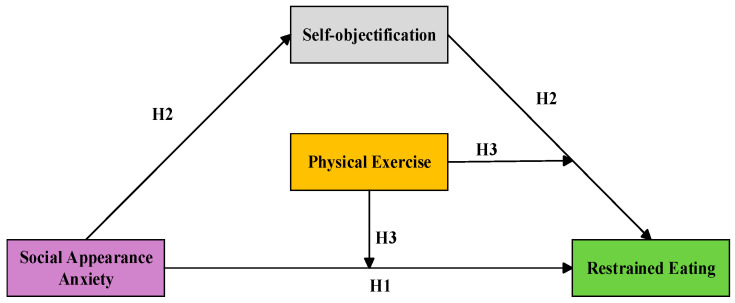
The mediating role of self-objectification and the moderating role of physical exercise in the relationship between social appearance anxiety and dietary restrictions.

The findings are expected to clarify how these factors interact specifically among female college students. Such insights can help design interventions to reduce their SAA and RE, thereby promoting their overall physical and mental development. The hypothesized model is shown in Figure 1.

## 3. Materials and Methods

### 3.1. Participants and Procedure

We conducted a cross-sectional online survey. A priori power analysis was performed using G*Power 3.1 to determine the minimum sample size. For a moderated mediation model estimated with multiple regression, results indicated that 193 participants were required to detect a medium effect size (f^2^ = 0.15) at a significance level of 0.05 with 80% power (Kang, 2021). Considering that the model included five predictors, and following the rule of thumb of 10–15 participants per questionnaire item, an adequate sample size was estimated to be between 350 and 525. On this basis, we set a target of at least 350 participants (Lakens, 2021).

A total of 2238 questionnaires were collected via an online platform, with each IP address restricted to a single submission to preserve data integrity. Invalid responses (n = 21) were excluded if completed in implausibly short times or contained invariant answers. Furthermore, surveys with absolute skewness > 1 or kurtosis > 3 were considered outliers and removed (n = 56). After screening, 2161 valid questionnaires remained, yielding a 96.6% valid response rate. To minimize missing data, all items were set as mandatory and underwent real-time validation on the platform; thus, the final dataset contained no missing values. Robustness was further assessed through sensitivity analyses using (a) the full dataset (n = 2238) and (b) alternative trimming thresholds (absolute skewness > 2, kurtosis > 7). The findings were consistent across all specifications.

All participants received a small incentive of ¥2 (≈$0.28) via electronic transfer. They were fully informed about the study objectives, confidentiality protections, and their right to withdraw at any time. Ethical approval was granted by the Institutional Review Board of Jiangxi Normal University (IRB-JXNU-PEC-20240104), and all procedures complied with the Declaration of Helsinki.

### 3.2. Measures

#### 3.2.1. Social Appearance Anxiety

We used the Social Appearance Anxiety Scale (Hart et al., 2008) to assess individuals’ anxiety about their physical appearance in social contexts. The scale consists of 16 items rated on a 5-point Likert scale (1 = not at all characteristic of me to 5 = extremely characteristic of me), with higher scores indicating higher levels of social appearance anxiety. Item 1 is reverse-scored. A sample item is: “I worry that others will judge my looks in social situations.” In the present study, the SAAS demonstrated good internal consistency, with a Cronbach’s α of 0.872. Furthermore, confirmatory factor analysis indicated that the scale showed good structural validity (χ^2^/df = 1.388, CFI = 0.995, TLI = 0.944, RMSEA = 0.013), supporting the reliability and validity of the Social Appearance Anxiety Scale in this study.

#### 3.2.2. Self-Objectification

Self-objectification was assessed with the Body Surveillance Scale, a subscale of the Objectified Body Consciousness Scale (McKinley & Hyde, 1996), revised by Sun (2018) for the target population. The scale includes 8 items rated on a 5-point Likert scale, with higher scores indicating greater self-objectification. In this study, the Body Surveillance Scale showed good internal consistency (Cronbach’s α = 0.826) and structural validity (χ^2^/df = 1.833, CFI = 0.996, TLI = 0.995, RMSEA = 0.020).

#### 3.2.3. Restrained Eating

We assessed restrained eating behavior in female college students using the Restrained Eating Subscale of the Dutch Eating Behavior Questionnaire (Van Strien et al., 1986), which was adapted into Chinese by S. Wu et al. (2017). This subscale contains 10 items rated on a 5-point Likert scale, with higher scores reflecting greater restrained eating tendencies. A sample item is: “Do you take into account your weight with what you eat?” In the present study, the subscale demonstrated good internal consistency (Cronbach’s α = 0.814). Moreover, confirmatory factor analysis indicated that the measurement model of restrained eating showed good structural validity (χ^2^/df = 1.668, CFI = 0.994, TLI = 0.993, RMSEA = 0.018), supporting the reliability and validity of this instrument in the current research.

#### 3.2.4. Physical Exercise

Physical exercise was measured with a single question: “Over the past 7 days, how many days did you engage in at least 20 min of PE or activity that made you sweat or breathe heavily?” Participants could respond with a number ranging from 0 to 7 days. This assessment method has been used in previous studies (Roberts et al., 2024; Uddin et al., 2020; Wang et al., 2025). Previous studies with female college students have also employed this item (W. Huang et al., 2025b), supporting its applicability in this population.

### 3.3. Data Analysis

We used SPSS 26.0 for data entry, organization, and descriptive statistical analysis. To assess the measurement model, confirmatory factor analysis (CFA) was conducted using AMOS 28.0, following established recommendations for structural validity testing. To test the mediating role of SO and the moderating role of PE, we applied the PROCESS macro (version 3.5) (Hayes et al., 2017) in SPSS. After controlling for variables such as age, education level, place of birth, ethnicity, and BMI, we constructed a moderated mediation model where SAA served as the predictor variable, RE as the outcome variable, SO as the mediator, and PE as the moderator. For the mediation analysis, we used Model 4 and the bias-corrected bootstrap method with 5000 resamples to calculate the 95% confidence interval (CI), with significance indicated by a CI excluding zero (Cheung & Lau, 2008). For the moderation analysis, we used Model 15, standardizing variables before computing interaction terms.

## 4. Results

### 4.1. Common Method Bias Test

To address the potential issue of common method bias arising from female college students’ self-reports, we conducted both Harman’s single-factor test and a CFA following the recommendations of Podsakoff et al. (2003). The results of Harman’s single-factor test indicated that three factors had eigenvalues greater than 1, and the first factor explained 24.089% of the variance, which is below the critical threshold of 40%. Furthermore, when all variables were constrained to load onto a single latent factor in CFA, the model fit was poor (χ^2^/df = 10.185, CFI = 0.724, TLI = 0.707, RMSEA = 0.065), further suggesting that no single factor accounted for the majority of covariance among the measures. Together, these results indicate that common method bias was not a serious concern in the present study.

### 4.2. Sample Characteristics

The sample characteristics are presented in Table 1, both for the total sample and segmented by education level, place of birth, and ethnicity. The final sample included 2161 female college students aged 18–31 years (M = 20.47, SD = 1.93). The majority were undergraduates (88.5%), with smaller proportions of master’s (10.4%) and doctoral students (1.1%). Slightly more than half of the respondents were from urban areas (55.8%), while 44.2% were from rural areas. In terms of ethnicity, 77.7% were Han and 22.3% were minority. Regarding BMI, 23.0% were underweight, 53.4% were normal weight, 17.7% were overweight, and 5.9% were obese.

Group comparisons revealed several significant differences. Independent samples t-tests indicated significant differences in RE and SAA by place of birth, and in SAA and PE by ethnicity. One-way ANOVAs further showed significant differences in SO, RE, and PE across education levels, and in SAA, RE, and PE across BMI categories. Based on these findings, all subsequent analyses controlled for place of birth, ethnicity, education, and BMI. See Table 1.

### 4.3. Correlation Results

Table 2 displays the descriptive statistics and relationships among the variables. SAA is positively associated with RE and SO, while SO is negatively related to PE and positively to RE. PE is inversely related to both SO and RE.

### 4.4. The Impact of Social Appearance Anxiety on Restrained Eating

We used hierarchical regression analysis to examine the effect of SAA on RE while controlling for education level, place of birth, ethnicity, and BMI. The results (Table 3) show that SAA is significantly positively related to RE (*β* = 0.467, *p* < 0.001). This suggests that higher levels of SAA are associated with a substantial increase in RE, thereby confirming Hypothesis 1.

### 4.5. The Mediation Analyses

**Figure 2:**
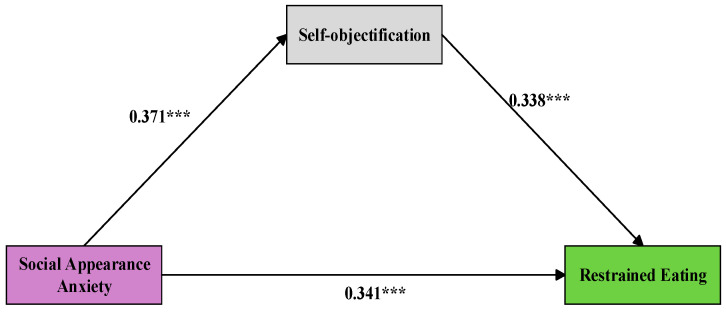
Indirect effect of social appearance anxiety on eating restriction through self-objectification, *** Significant correlation at 0.001 level.

We used the Variance Inflation Factor (VIF) to check for multicollinearity among the variables. The results showed that the highest VIF value was 1.317, well below the commonly used threshold of 10, indicating no serious multicollinearity among the variables (Marcoulides & Raykov, 2019). After standardizing the variables, we used Model 4 from the SPSS macro developed by Hayes to test the mediating effect of SO. Controlling for education level, place of birth, ethnicity, and BMI, we found that SAA significantly predicts SO (*β* = 0.371, *t* = 18.500, *p* < 0.001) and also predicts with RE (*β* = 0.341, *t* = 17.862, *p* < 0.001). Additionally, SO significantly predicts with RE (*β* = 0.338, *t* = 17.743, *p* < 0.001). The mediating effect value was 0.125, and the Bootstrap 95% confidence interval was [0.104, 0.149]. This mediating effect, while statistically significant, may only account for a portion of the variance in the relationship between SAA and RE, supporting Hypothesis 2 (See Figure 2).

### 4.6. Moderated Mediation Models

We used SPSS Process Model 15 to test the moderated mediation model. After controlling for education level, place of birth, ethnicity, and BMI, we analyzed the model with SO as the mediator. The detailed results are shown in Table 4. Because all variables were standardized prior to analysis, the coefficients reported below are standardized estimates. When we added PE to the model, the interaction between SAA and PE was significantly related to RE (*β* = −0.152, *t* = −10.095, *p* < 0.001, 95% CI = [−0.182, −0.122]). However, the interaction between SO and PE was not significant (*β* = 0.015, *p* = 0.395, 95% CI = [−0.019, 0.049]). This indicates that PE moderates the relationship between SAA and RE, but only on the direct pathway. In contrast, the indirect pathway via SO was not moderated by PE. Thus, Hypothesis 3 is only partially supported.

**Figure 3:**
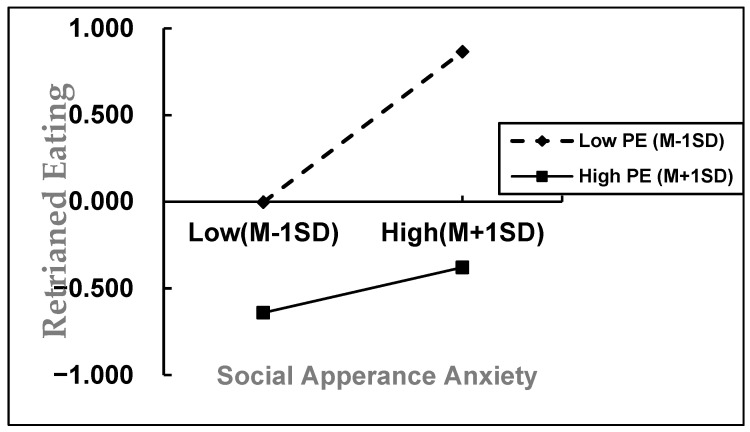
The moderating effect of physical exercise on social appearance anxiety and restrained eating.

To better explain the moderated mediation model, we divided participants into high and low PE groups based on one standard deviation above or below the mean level of PE. We then used simple slopes analysis to examine how PE affects the relationship between SAA and RE. Figure 3 presents the simple slopes analysis, illustrating the relationship between SAA and RE. The results show that in the low PE group, SAA has a stronger effect on RE (*β* = 0.435, *t* = 18.447, *p* < 0.001). However, in the high PE group, this effect is weaker (*β* = 0.131, *t* = 6.165, *p* < 0.001). This means that as PE increases, the effect of SAA on RE gets smaller.

Given that the median number of PE days was 2, we also conducted a robustness check by dichotomizing PE into a low PE group (≤2 days/week) and a high PE group (≥3 days/week). The moderation analysis yielded results consistent with those reported above: the SAA × PE interaction remained significant (*β* = −0.355, *p* < 0.001, 95% CI = [−0.419, −0.292]). Simple slope analysis further confirmed that the effect of SAA on RE was stronger in the low PE group than in the high PE group, corroborating the buffering role of PE.

## 5. Discussion

### 5.1. Social Appearance Anxiety and Restrained Eating

The results of this study indicate that SAA is positively correlated with RE among female college students, confirming Hypothesis 1. Higher levels of SAA were linked to a greater likelihood of engaging in restrictive eating behaviors. This finding aligns with prior studies showing that persistent negative self-evaluation and frequent appearance comparisons can contribute to maladaptive cognitive patterns, body dissatisfaction, and, in turn, eating disorders, creating a detrimental cycle (Brosof & Levinson, 2017; Caner et al., 2022; Cataldo et al., 2022; Fu et al., 2022; Rodgers et al., 2020; Vandenbosch et al., 2022). SAA, as conceptualized by social comparison theory, arises from individuals’ tendency to evaluate themselves against others (Dijkstra et al., 2008; Gerber et al., 2018). In contemporary contexts saturated with idealized appearance images on social media, this tendency may be particularly pronounced (Deng & Jiang, 2023; Fox et al., 2021; Maheux et al., 2021; Veldhuis et al., 2020; Xian et al., 2024). Striving for such ideals often intensifies, rather than relieves, appearance-related anxiety, thereby elevating the risk of disordered eating. Overall, these findings suggest that SAA may play an important role in shaping eating behaviors among female college students and valuable reference for future studies

### 5.2. Self-Objectification as a Mediator

This study found that SO partially mediates the relationship between SAA and RE, confirming Hypothesis 2. This suggests that SAA not only has a direct impact on RE but also exerts an indirect effect through heightened SO. From a social cognitive perspective, repeated negative self-evaluations triggered by SAA weaken perceived self-efficacy in appearance management, thereby reinforcing SO (Daniels et al., 2020; Salomon & Brown, 2021; Schaefer & Thompson, 2018).

In line with objectification theory, SO substantially influences cognition, motivation, emotion, and behavior (Fredrickson et al., 1998; Szymanski & Henning, 2007). Elevated SO encourages individuals to constantly monitor their appearance against external standards (Y. Wu et al., 2024) and to reinterpret appearance-related situations as uncontrollable stressors rather than manageable challenges. As a result, the body is experienced as an object requiring continual improvement (Veldhuis et al., 2020), leading to maladaptive coping in the form of restrained eating rather than healthier strategies.

Furthermore, guilt-related processes may interact with SO and help explain the persistence of RE, underscoring the complexity of these psychological pathways (Raffone et al., 2025). In everyday contexts, high SO is associated with rigid and inefficient regulatory strategies, which intensify negative emotions, suppress positive expression, and worsen mental health (Schaefer et al., 2018; Skowronski et al., 2021). Taken together, the findings highlight SO as a central pathway linking SAA and RE and suggest that interventions aimed at reducing SO may help mitigate the risk of disordered eating behaviors.

### 5.3. Physical Exercise as a Moderator

The results indicate that PE significantly moderates the relationship between SAA and RE behaviors, but not the relationship between SO and RE, thereby partially confirming Hypothesis 3. Specifically, individuals with higher PE participation showed lower levels of RE even when experiencing high SAA, compared to those with lower PE participation. This finding supports research in sports psychology suggesting that engagement in PE not only elevates mood and cognitive functioning but also serves as a protective factor against mental health risks (S. Biddle, 2016; S. J. H. Biddle et al., 2019; Lubans et al., 2016; Mahindru et al., 2023). By fostering positive experiences such as vitality and a more optimistic outlook, PE may help individuals cope more effectively with appearance-related anxiety (Lee et al., 2025; Ruiz-Ranz & Asín-Izquierdo, 2024; White et al., 2023). Importantly, these benefits shift attention from outward appearance toward physical competence, thereby reducing the translation of SAA into restrictive eating behaviors (Skowronski et al., 2021; Zheng et al., 2018). This mechanism is consistent with integrative performance models, which highlight how positive psychological experiences during PE—encompassing thoughts, emotions, and bodily sensations—can strengthen overall well-being (Dores et al., 2021; L. Liu et al., 2024).

At the same time, the study lends support to the exclusion hypothesis of the protective model (Fergus & Zimmerman, 2005; Zimmerman et al., 2013), indicating that PE can buffer the impact of SAA on RE by diminishing its negative influence (Reigal et al., 2020). However, the absence of moderation in the SO–RE pathway requires further reflection. Unlike SAA, which reflects a more situational and transient form of anxiety, SO reflects a stable cognitive orientation in which individuals habitually monitor and evaluate their bodies from an external perspective. This chronic self-surveillance exerts influence not only through heightened body dissatisfaction but also through internalized social standards, shame, and inadequacy. Because these mechanisms are deeply ingrained in self-concept and emotional regulation, they may be resistant to the short-term psychological benefits of PE. Put differently, while PE appears to buffer temporary appearance-related anxiety, it is less effective in counteracting the more enduring and pervasive effects of SO on disordered eating (Fietz et al., 2015; Li et al., 2024).

### 5.4. Recommendation

To address the prevalent SAA and RE among female college students, society, schools, and families need to enhance their awareness of the impact of these phenomena on college students’ emotions, behaviors, and physical and mental health. The results of this study show that SAA is significantly associated with RE behavior, and it is necessary to take measures to mitigate the long-term impact of this anxiety on college students.

Firstly, at the societal level, media regulation should be strengthened to reduce the promotion of a singular aesthetic standard and promote a more diverse range of aesthetic views, thereby effectively lowering the impact of SAA on college students. Schools and communities should also take proactive steps to organize more educational activities focused on mental health and body positivity, helping college students develop a healthy self-image and eating habits. Research has shown that mindfulness interventions can effectively reduce SAA, thereby decreasing the incidence of RE behavior (Seekis et al., 2020; Zoogman et al., 2015).

Secondly, schools and counseling centers can offer workshops and support groups to address the impact of media and social comparisons on body image and self-esteem. These programs should reduce the internalization of unrealistic beauty standards and promote a healthy body image. Educational institutions can integrate promoting healthy body image and reducing SO into their mental health education guidelines and wellness programs, offering courses or seminars on media literacy, body positivity, and the impact of social media on mental health.

Lastly, in terms of PE, physical education programs should emphasize the importance of PE for mental health and wellbeing. Universities can provide a variety of physical activities, such as structured group-based sports tailored to the interests and needs of female college students, like campus Latin dance. They can also develop comprehensive fitness programs offering diverse exercise options, including yoga, Pilates, high-intensity interval training, and strength training. These findings highlight the potential value of incorporating body positivity programs that address multiple risk pathways, yet replication of the present results is required before such interventions are broadly applied.

### 5.5. Limitations

While this study has made some progress in exploring the impact of SAA on RE behavior among female college students and its underlying mechanisms, there are still some limitations. First, the study adopted a cross-sectional design, so the results only indicate associations among the variables rather than causal relationships. Causal inferences regarding the directions of these associations should be made with caution. Future research could also employ longitudinal designs to examine how SAA, SO, PE, and RE change over time, which would help clarify the temporal order of these associations. In addition, experimental studies, such as manipulating exposure to appearance-related media or implementing structured exercise interventions, could provide stronger evidence for potential causal mechanisms.

Second, the study sample is limited to Chinese female college students from only two provinces (Yunnan and Guizhou), and participants were recruited through convenience sampling. Nearly 90% were undergraduates, while the proportions of master’s and doctoral students were much lower. These characteristics may introduce selection bias and restrict the generalizability of the findings to female college students in other regions, at different educational stages, or in broader populations. In addition, all data were self-reported, which may result in common method bias or social desirability effects. However, Harman’s single-factor test suggested that such bias was not a major concern in this study. Future research should seek to broaden the sample to individuals with diverse ages, genders, occupations, and cultural backgrounds, and conduct cross-cultural comparisons to improve the universality of the findings. The use of objective measures or multiple data sources would also help reduce the limitations associated with self-reports.

Third, this study assessed physical exercise using a single self-report item. Although this approach is convenient, it cannot fully capture the quality, intensity, or nature of activity (e.g., distinctions between structured exercise and recreational activity). To test the robustness of our findings, we conducted a median split analysis (low PE ≤ 2 days/week; high PE ≥ 3 days/week). The moderation effect of PE on the SAA–RE pathway remained significant, thereby supporting the stability of our results. Nevertheless, relying on a single-item indicator restricts measurement to exercise frequency and entails clear limitations. Although this item has previously been applied to female college student populations, future studies should adopt validated, multidimensional instruments to provide a more comprehensive understanding of physical activity and its moderating role.

Lastly, although this study primarily examines the effects of SAA, SO, and PE on RE behavior, the development of RE behavior is a complex process that may also be influenced by other factors, such as family environment, socio-cultural background, and individual psychological traits. Future research could further investigate the interactions between these factors and SAA, SO, and PE, as well as their combined impact on RE.

## 6. Conclusions

RE is a common eating behavior that can lead to various physical and mental health issues. This study has deeply explored the roles of SO and PE in the relationship between SAA and RE. The results show that SO plays a mediating role in the relationship between SAA and RE, while PE acts as a moderator. Specifically, SAA has a positive impact on RE through the mediating effect of SO. In addition, PE serves as a protective factor, reducing the impact of SAA on RE. These findings highlight the importance of considering both cognitive (SO) and behavioral (PE) factors in understanding disordered eating behaviors.

## Figures and Tables

**Table 1:** Demographic characteristics and group differences.

Variables	N (%)	*t*/*F*-Value	SAA Mean (SD)	SO Mean (SD)	RE Mean (SD)	PE Mean (SD)
Education						
Undergraduate	1912 (88.5%)		2.97 (0.83)	2.84 (0.98)	2.92 (0.81)	2.47 (2.13)
Master’s student	225 (10.4%)		2.90 (0.77)	2.72 (0.93)	2.73 (0.75)	2.51 (2.25)
Doctoral student	24 (1.1%)		2.69 (1.44)	2.05 (1.08)	2.33 (1.14)	4.46 (2.45)
		*F*-value	1.765	8.846 ***	11.311 ***	10.187 ***
Place of birth						
Urban	1205 (55.8%)		3.01 (0.91)	2.81 (1.03)	2.94(0.88)	2.48 (2.20)
Rural	956 (44.2%)		2.89 (0.73)	2.81 (0.73)	2.84(0.72)	2.51 (2.10)
		*t*-value	3.191 **	0.230	2.868 **	−0.330
Ethnicity						
Han	1680 (77.7%)		2.98 (0.86)	2.82 (1.00)	2.89 (0.83)	2.55 (2.17)
Minority	481 (22.3%)		2.88 (0.76)	2.81 (0.93)	2.92 (0.75)	2.31 (2.10)
		*t*-value	2.529 *	0.194	−0.654	2.111 *
BMI						
<18.5	497 (23.0%)		2.92 (0.81)	2.88 (0.91)	2.88 (0.75)	2.40 (2.05)
18.5~23.9	1153 (53.4%)		3.03 (0.90)	2.78 (1.08)	2.95 (0.90)	2.63 (2.28)
24~27.9	383 (17.7%)		2.87 (0.69)	2.84 (0.80)	2.82 (0.66)	2.38 (1.98)
>28	128 (5.9%)		2.71 (0.64)	2.82 (0.73)	2.69 (0.54)	1.98 (1.77)
		*F*-value	8.268 ***	1.263	5.348 **	4.813 **

Note: SAA (Social Appearance Anxiety), SO (Self-objectification), RE (Restrained Eating), PE (Physical Exercise). * Significantly correlated at the 0.05 level, ** Significantly correlated at the 0.01 level, *** Significantly correlated at the 0.001 level (two-tailed).

**Table 2:** Variable Statistics and Relationships.

Variables	M	SD	Skewness	Kurtosis	1	2	3	4
1. SAA	2.958	0.836	0.034	−0.125	1			
2. SO	2.814	0.981	−0.071	−0.678	0.371 **	1		
3. RE	2.895	0.814	0.261	−0.211	0.471 **	0.469 **	1	
4. PE	2.497	2.153	0.561	−0.948	−0.298 **	−0.417 **	−0.606 **	1

Note: SAA (Social Appearance Anxiety), SO (Self-objectification), RE (Restrained Eating), PE (Physical Exercise). ** Significant at 0.01 level (two-tailed). M: mean. SD: standard deviation.

**Table 3:** Hierarchical regression analysis of factors influencing restrained eating.

Model	Variables	Restrained Eating			
		*β*	*SE*	*F*	*R* ^2^
1	Education	−0.101 ***	0.048	8.980 ***	0.015
	Place of birth	−0.062 **	0.035		
	Ethnicity	0.015	0.042		
	BMI	−0.047 *	0.022		
2	Education	−0.083 ***	0.042	129.936 ***	0.232
	Place of birth	−0.031	0.031		
	Ethnicity	0.039 *	0.037		
	BMI	−0.024	0.019		
	Social Appearance Anxiety	0.467 ***	0.018		

Note: * Significant at 0.05 level, ** Significant at 0.01 level, *** Significant at 0.001 level.

**Table 4:** Moderated mediation regression results.

Variables	Self-Objectification		Restrained Eating	
	*β*	*t*	*β*	*t*
Education	−0.168	−3.054 **	−0.149	−3.602 **
Place of birth	0.043	1.058	−0.044	−1.441
Ethnicity	0.040	0.827	0.040	1.094
BMI	0.010	0.411	−0.048	−2.552 *
social appearance anxiety	0.371	18.500 ***	0.283	17.032 ***
Self-objectification			0.176	10.171 ***
social appearance anxiety × physical exercise			−0.152	−10.095 ***
Self-objectification × physical exercise			0.015	0.851
F	71.357		254.804	
R^2^	0.142		0.516	

Note. * Significant correlation at 0.05 level, ** Significant correlation at 0.01 level, *** Significant correlation at 0.001 level.

## Data Availability

The data used in this study are available from the corresponding author upon reasonable request.

## Abbreviations

The following abbreviations are used in this manuscript:

SAA	Social Appearance Anxiety
SO	Self-objectification
RE	Restrained Eating
PE	Physical Exercise
CFA	Confirmatory Factor Analysis
CI	Confidence Interval
BMI	Body Mass Index
VIF	Variance Inflation Factor
